# Probiotic Lactobacilli Modulate *Staphylococcus aureus*-Induced Activation of Conventional and Unconventional T cells and NK Cells

**DOI:** 10.3389/fimmu.2016.00273

**Published:** 2016-07-11

**Authors:** Maria A. Johansson, Sophia Björkander, Manuel Mata Forsberg, Khaleda Rahman Qazi, Maria Salvany Celades, Julia Bittmann, Matthias Eberl, Eva Sverremark-Ekström

**Affiliations:** ^1^Arrhenius Laboratories for Natural Sciences, Department of Molecular Biosciences, The Wenner-Gren Institute, Stockholm University, Stockholm, Sweden; ^2^Division of Infection and Immunity, School of Medicine, Cardiff University, Cardiff, UK; ^3^Systems Immunity Research Institute, Cardiff University, Cardiff, UK

**Keywords:** cell-free supernatant, immune modulation, lactobacilli, NK cells, probiotic, T cells, *Staphylococcus aureus*, superantigens

## Abstract

Lactobacilli are probiotic commensal bacteria and potent modulators of immunity. When present in the gut or supplemented as probiotics, they beneficially modulate *ex vivo* immune responsiveness. Further, factors derived from several lactobacilli strains act immune regulatory *in vitro*. In contrast, *Staphylococcus aureus* (*S. aureus*) is known to induce excessive T cell activation. In this study, we aimed to investigate *S. aureus*-induced activation of human mucosal-associated invariant T cells (MAIT cells), γδ T cells, NK cells, as well as of conventional CD4^+^ and CD8^+^ T cells *in vitro*. Further, we investigated if lactobacilli-derived factors could modulate their activation. PBMC were cultured with *S. aureus* 161:2 cell-free supernatants (CFS), staphylococcal enterotoxin A or CD3/CD28-beads alone, or in combination with *Lactobacillus rhamnosus* GG-CFS or *Lactobacillus reuteri* DSM 17938-CFS and activation of T and NK cells was evaluated. *S. aureus*-CFS induced IFN-γ and CD107a expression as well as proliferation. Costimulation with lactobacilli-CFS dampened lymphocyte-activation in all cell types analyzed. Preincubation with lactobacilli-CFS was enough to reduce subsequent activation, and the absence of APC or APC-derived IL-10 did not prevent lactobacilli-mediated dampening. Finally, lactate selectively dampened activation of unconventional T cells and NK cells. In summary, we show that molecules present in the lactobacilli-CFS are able to directly dampen *in vitro* activation of conventional and unconventional T cells and of NK cells. This study provides novel insights on the immune-modulatory nature of probiotic lactobacilli and suggests a role for lactobacilli in the modulation of induced T and NK cell activation.

## Introduction

Lactobacilli are commensal, lactic acid-producing bacteria with proven beneficial effects when used as dietary supplements or when present in the early neonatal gut. Lactobacilli appear to protect against certain immune-mediated diseases (1, 2) and have been suggested to act as important immune modulators, especially during early life. Indeed, different lactobacilli strains can modulate stimulated responses *in vitro* (3, 4) and supplementation with different strains of lactobacilli is associated with dampened *ex vivo* PBMC responsiveness (5). In contrast, *Staphylococcus aureus* (*S. aureus*) is a pathobiont and a common colonizer of the infant gut (1, 6). *S. aureus* is a potent immune activator as superantigenic staphylococcal enterotoxins (SE) are able to engage large numbers of conventional T cells *via* MHC-mediated binding to the variable domain of the T cell receptor (TCR) β-chain (7) or α-chain (8). It thereby efficiently avoids immune clearance by inducing T cell exhaustion and anergy (9). Despite obvious effects on the lymphocyte compartment as a whole, the effect of *S. aureus* and its enterotoxins on unconventional T cells and NK cells has not been extensively studied (10).

We have previously shown that colonization with *S. aureus* in early life is associated with increased PBMC cytokine-secretion at the age of two, while co-colonization with lactobacilli results in dampened immune reactivity *in vitro* (11), findings that together further support the hypothesis that lactobacilli are involved in immune regulation *in vivo*. In addition, we reported that soluble factors derived from lactobacilli dampen *S. aureus*-induced activation of CD4^+^ T cells on a cellular level and also reduce the release of T cell-associated pro-inflammatory cytokines (12), showing that lactobacilli can exert immune dampening effects on selected compartments of the immune system.

Lactobacilli mediate their immune-modulatory effects through the induction of regulatory cytokines, such as IL-10 (13, 14), induction of T regulatory cells (15–17), modulation of APC (18–20), promotion of epithelial function and development (21), and by inhibition of pro-inflammatory cytokines (22, 23). Extracted cell surface components of lactic acid bacteria induce a more pro-inflammatory cytokine profile, whereas cell-free supernatants (CFS) are more prone to induce an anti-inflammatory response by PBMC (24). The induction of pro-inflammatory responses seen with whole lactobacilli *in vitro* might not appropriately reflect peripheral immune cell modulation *in vivo*, as whole bacteria will most likely not enter the blood stream in large numbers. Instead, bacterial metabolites have been shown to cross the epithelial barrier, retain their bioactive properties, and affect peripheral immunity *in vitro* and *in vivo* (24–26). Secreted factors produced by lactobacilli have been extensively examined and factors, such as p40 and histamine, are discussed as potential effector molecules (27, 28). However, if, and by which mechanisms, lactobacilli-derived molecules are able to mediate immune-modulatory effects on lymphocytes, is largely unknown.

Here, we investigated *S. aureus* and enterotoxin-mediated activation of T cells and NK cells, and how lactobacilli-derived factors dampen *S. aureus*-induced activation *in vitro*. We show that soluble factors, including enterotoxins, derived from *S. aureus* 161:2 potently induce several effector functions in unconventional T cells and NK cells, in addition to conventional T cells. Soluble factors derived from two common probiotic lactobacilli strains, *Lactobacillus rhamnosus* (*L. rhamnosus*) GG (LGG) and *Lactobacillus reuteri* DSM 17938 (*L. reuteri*), were able to directly dampen *S. aureus*-induced lymphocyte-activation *in vitro*, without the involvement of APC or APC-derived IL-10. This *in vitro* study provides a possible link to the immune-modulatory capacity of lactobacilli *in vivo* and suggests that lactobacilli can modulate pathogen-induced immune activation.

## Materials and Methods

### Subjects, Ethics Statement, and Isolation of Peripheral Blood Mononuclear Cells

A total of 18 healthy, anonymous, adult volunteers (age 18–65, both genders) were included in this study, which was approved by the Regional Ethic’s Committee at the Karolinska Institute, Stockholm, Sweden [Dnr 04-106/1 and 2014/2052-32]. All study subjects gave their informed written consent. Venous blood was collected in heparinized vacutainer tubes (BD Biosciences Pharmingen) and diluted with RPMI-1640 supplemented with 20 mM HEPES (HyClone Laboratories, Inc.). PBMC were isolated by Ficoll-Hypaque (GE Healthcare Bio-Sciences AB) gradient separation. The cells were washed and resuspended in freezing medium containing 40% RPMI-1640, 50% FCS (Life Technologies), and 10% DMSO, gradually frozen in a freezing container (Mr. Frosty, Nalgene Cryo 1°C; Nalge Co.) and stored in liquid nitrogen.

### Strains of Bacteria and Generation of Bacterial Cell-Free Supernatants

*L. rhamnosus* GG (ATCC 53103; isolated from the probiotic product Culturelle), *L. reuteri* (DSM 17938, a gift from Biogaia AB), and *S. aureus* 161:2 (carrying the genes for SE A and H) were the species used in this study. The *S. aureus* strain was a kind gift from Åsa Rosengren, The National Food Agency, Uppsala, Sweden, who screened the strain for toxin genes by using PCR. The lactobacilli were cultured in MRS broth (Oxoid) at 37°C for 20 h and *S. aureus* in BHI broth (Merck) at 37°C for 72 h still culture. The bacteria were pelleted by centrifugation at 3400 *g*, and the CFS were sterile filtered (0.2 μm) and frozen at −20°C until used. All bacterial supernatants were diluted 1:1 (50%) with HEPES to neutralize the pH. The final CFS concentration used for cell stimulations was 2.5%, unless stated otherwise. To investigate heat stability, the LGG-CFS was heat-treated for 10 min by boiling at 100°C.

### *In Vitro* Stimulation of PBMC

PBMC were thawed and washed before being counted and viability assessed by Trypan blue staining. Cells were resuspended to a final concentration of 1 × 10^6^ cells/ml in cell culture medium (RPMI-1640 supplemented with 20 mM HEPES), penicillin (100 U/ml), streptomycin (100 μg/ml), l-glutamine (2 mM) (all from HyClone Laboratories Inc.), and 10% FCS (Life Technologies). The cells were seeded in flat-bottomed 48-well plates (Costar) for 24 h at 37°C in 5% CO_2_ atmosphere with cell culture medium alone as negative control or with Dynabeads Human T-Activator CD3/CD28 (Life Technologies) at 2:1 (cell:bead) ratio, 20 ng/ml of SEA (Sigma–Aldrich), or with *S. aureus* 161:2-CFS. As control, 50 ng/ml of PMA + 1 μg/ml of Ionomycin (IO) (both from Sigma–Aldrich) was used during the last 4 h of incubation. When indicated, LGG-CFS or *L. reuteri*-CFS was added together with the abovementioned stimuli during the entire incubation. Brefeldin A (BD Biosciences) was present during the last 4 h of incubation. For the preincubation experiments, PBMC were incubated with LGG-CFS or *L. reuteri*-CFS for 6 h and extensively washed with complete medium before being stimulated for 15 h.

### L(+)-Lactate Measurement and *In Vitro* Assay

The concentrations of L(+)-lactate in bacteria-CFS were quantified in four batches of CFS from each lactobacilli and in two batches of *S. aureus*-CFS using a colorimetric Lactate assay kit II (Sigma–Aldrich), according to the manufacturer’s instructions. In brief, samples were mixed with a reaction master mix and incubated for 30 min at room temperature after which absorbance was measured at 450 nm. Results were analyzed using SoftMax Pro 5.2 rev C (Molecular Devices Corp.). Neither the cell culture medium nor the bacterial growth medium interfered with the absorbance. To investigate the effect of l-lactate on *S. aureus*-mediated lymphocyte-activation, l-lactate (Sigma–Aldrich) was added to PBMC cultures at concentrations 0.75, 3.25, 7.5, or 38 mM (physiologically, levels in blood vary between 2 and 20 mM).

### Functional Assays

For measuring proliferation, PBMC were labeled at 37°C with 5 μM CellTrace™ Violet (Molecular Probes, Life Technologies), stimulated as described above, and cultured in complete medium at a concentration of 1 × 10^6^ cells/ml for 5 days. For assessing cytotoxic potential, PBMC were stimulated for 18 h in the presence of α-CD107a antibodies (clone: H4A39, BD Biosciences).

### IL-10 and IL-12 Neutralization

PBMC were seeded with an IL-10 neutralizing monoclonal antibody (clone: JES3-9D7, Biolegend) or matched isotype control (clone: RTK2071, Biolegend) at a concentration of 2.5 μg/ml for 2 h. PBMC were then stimulated and incubated overnight. Alternatively, PBMC were seeded with an IL-12 neutralizing monoclonal antibody (clone: MT3279H, Mabtech) or matched isotype control (clone: MPC-11, Biolegend) at 1 μg/ml for 5 h. PBMC were then stimulated, together with an additional dose of αIL-12/isotype control, for 24 h.

### CD14^+^ Monocyte Isolation and Depletion

The human CD14 positive selection kit (StemCell Technologies) was used to isolate or deplete CD14^+^ monocytes from PBMC according to the instructions of the manufacturer. CD14-depleted PBMC were cultured with 2.5% *S. aureus*-CFS in the presence or absence of LGG-CFS for 24 h with brefeldin A present during the last 4 h of incubation. Whole PBMC served as control. Isolated CD14^+^ monocytes were cultured at a concentration 0.5 × 10^6^/ml with 2.5% *L. reuteri*-CFS, 2.5% *S. aureus*-CFS, 100 ng/ml ultrapure LPS, or 10 μg/ml Pam3Cys (both Invivogen) for 14 h before culture supernatants were collected. Purity was assessed for all donors by staining with CD3 (clone: SK7) (Biolegend) and CD14 (clone: B159) (BD Biosciences). The mean percentage of CD14^+^ cells was 0.49 ± 0.28% in the monocyte-depleted PBMC and 97.4 ± 1.67% after purification.

### ELISA

Levels of IL-6, IL-10, IL-17A, and IFN-γ in cell culture supernatants were determined by using sandwich ELISA (MabTech AB) according to the instructions from the manufacturer. The optical density was determined using a microplate reader (Molecular Devices Corp.) set at 405 nm. Results were analyzed using SoftMax Pro 5.2 rev C (Molecular Devices Corp.).

### Flow Cytometry

After incubation, cells were harvested to V-shaped staining plates and washed in PBS. The cells were stained with the LIVE/DEAD Fixable Dead Cell Stain Kit-Aqua (Life Technologies) according to instructions from the manufacturer. Blocking of cell surface Fc receptors was done using 10% human serum in FACS wash buffer (PBS, 2 mM EDTA, and 0.1% BSA), and staining of cell surface markers was performed using the following antibodies: CD3 (clone: UCHT1, SK7), CD4 (clone: RPA-T4), CD8 (clone: SK1), CD25 (clone: M-A251), CD56 (clone: B159), CD127 (clone: HIL-7R-M21), CD161 (clone: HP-3G10), Vα7.2 (clone: 3C10) (BD Biosciences or Biolegend), and pan γδ TCR (clone: IMMU510) (Beckman Coulter). After surface staining, cells were washed with FACS wash buffer and fixed/permeabilized with the transcription factor buffer set (BD Biosciences) according to the manufacturer’s instructions. Intracellular blocking was done using 10% human serum, and cells were then stained for intracellular IFN-γ (clone: B27) or FoxP3 (clone: 2590/C7) (BD Biosciences). Stained cells were washed, resuspended, and acquired using a FACSVerse instrument and the FACS Suite software (both BD Biosciences). Lymphocytes were gated based on forward and side scatter properties. After gating on live cells, NK cells were gated as CD3^−^CD56^+^, T helper cells as CD3^+^CD4^+^, and T cytotoxic cells as CD3^+^CD8^+^. γδ T cells were classified as CD3^+^γδ TCR^+^, and MAIT cells were gated as CD3^+^CD161^+^Vα7.2^+^. Regulatory T cells were identified as CD4^+^CD25^+^FoxP3^+^CD127^low^. Unstimulated cells or corresponding isotype-matched antibodies were used as negative controls. Analyses were done with FlowJo Software (TreeStar).

### Statistics

GraphPad Prism software (GraphPad Software) was used for statistical analysis. For all analyses, the non-parametric, paired Wilcoxon matched pairs test was applied. Differences were considered significant if *p* < 0.05. The significance levels used were *p < 0.05, **p < 0.01, and ***p < 0.001.

## Results

### *S. aureus* 161:2-Induced IFN-γ Expression in T and NK Cells Is Partly IL-12-Dependent

**Figure 1:**
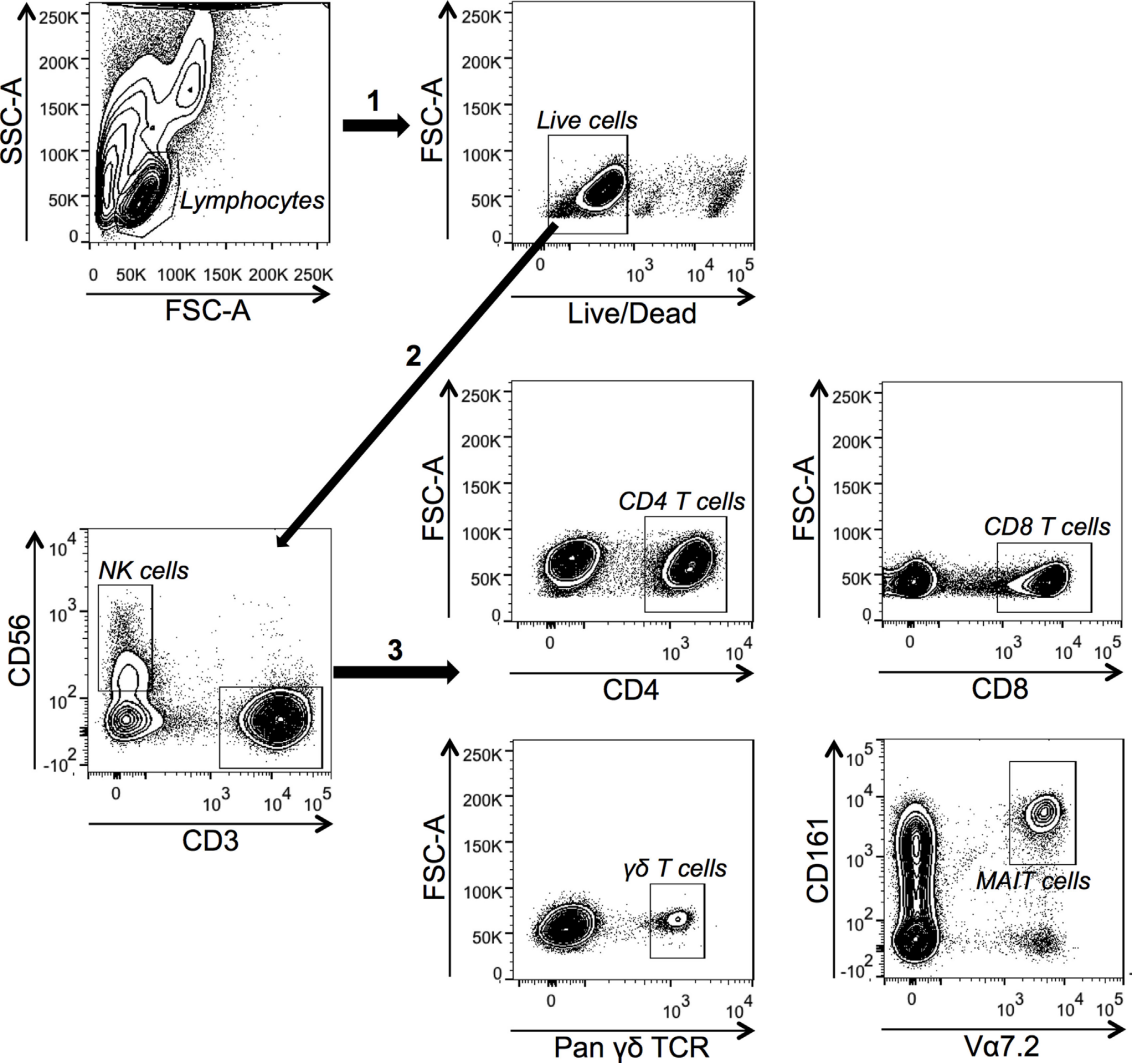
**Gating strategy used to identify T cells and NK cells by flow cytometry**. Lymphocytes were gated based on side scatter (SSC) and forward scatter (FSC) properties. Live cells (negatively stained for the Live/Dead-marker) were gated either as CD3^−^CD56^+^ cells (NK cells) or as CD3^+^CD56^−^ cells, which were further divided as CD4^+^ T cells, CD8^+^ T cells, pan γδ TCR^+^ cells (γδ T cells), or CD161^+^Vα7.2^+^ cells (MAIT cells). For all figures, gating strategies are described in the section “Materials and Methods.”

**Figure 2:**
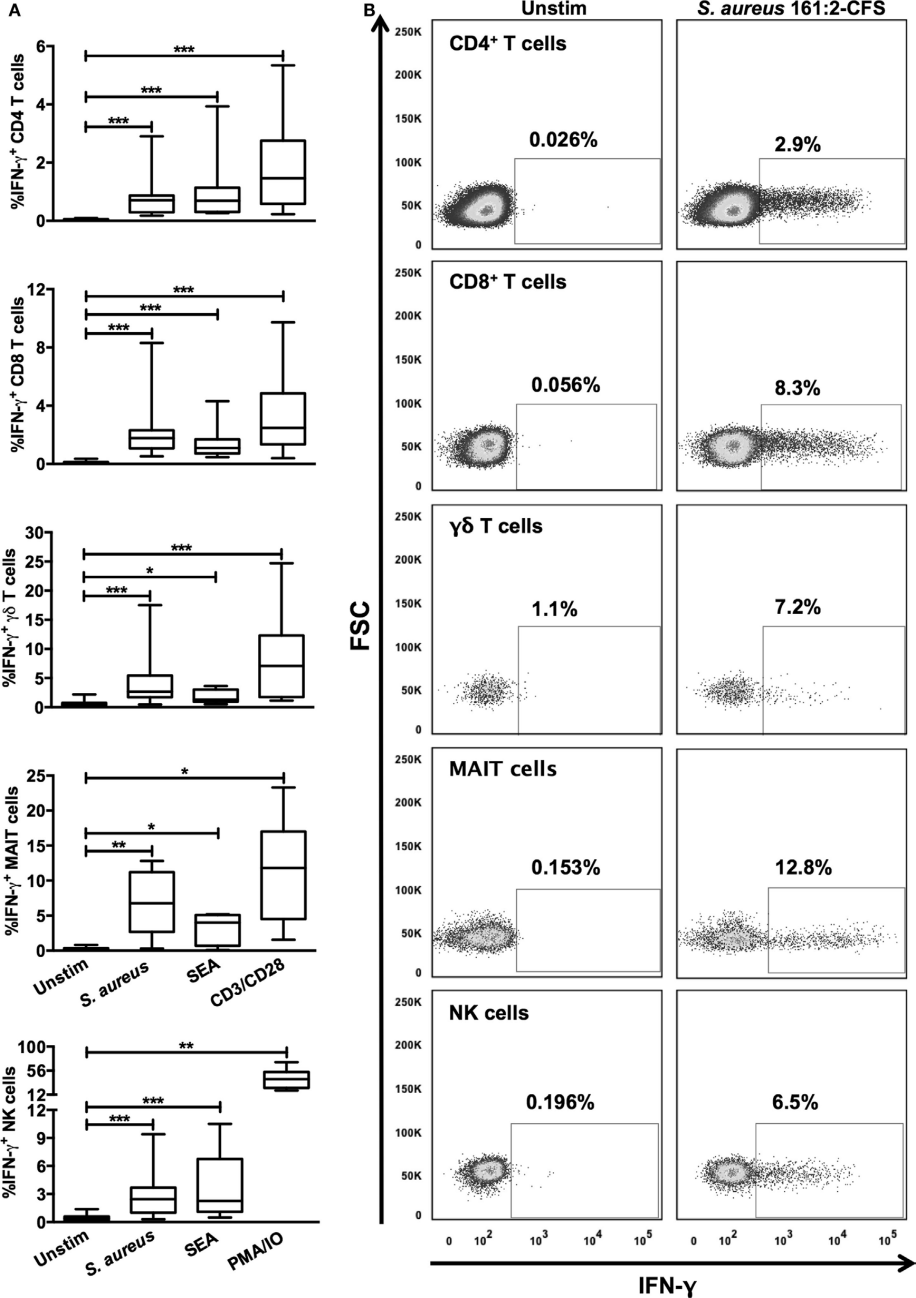
***S. aureus* 161:2 induces IFN-γ expression in T cells and NK cells**. PBMC were stimulated as indicated and analyzed by flow cytometry. **(A)** The percentage of IFN-γ^+^ cells among the CD4^+^ (*n* = 12–17), CD8^+^ (*n* = 12–18), γδ TCR^+^ (*n* = 7–15), CD161^+^Vα7.2^+^ (*n* = 6–7), and CD3^−^CD56^+^ (*n* = 8–17) lymphocyte populations is shown. **(B)** Examples of IFN-γ stainings of the cell populations described in **(A)**. Boxes cover data values between the 25th and 75th percentiles, with the central line as median and error bars showing minimal and maximal values.

**Figure 3:**
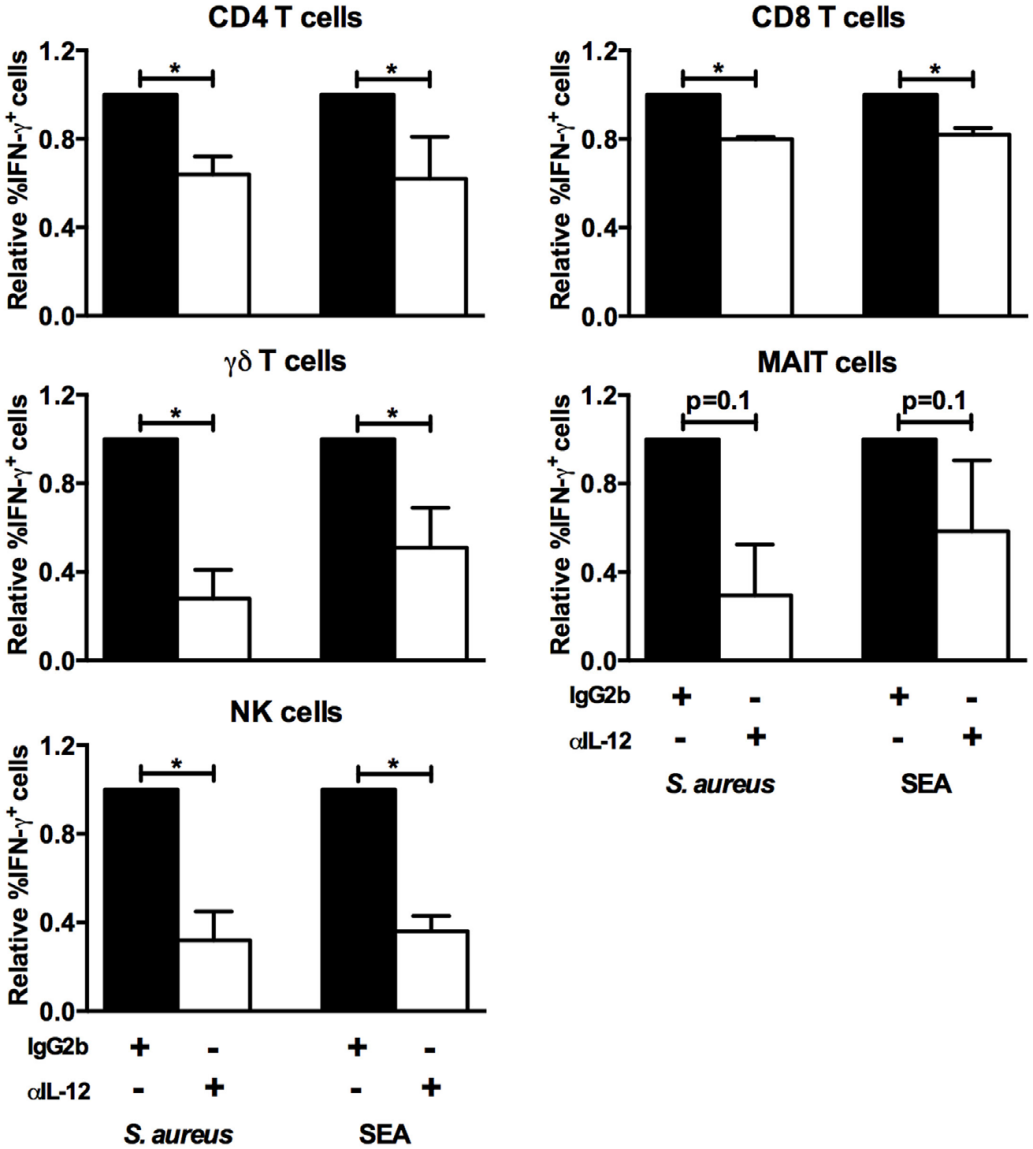
***S. aureus* and SEA-induced IFN-γ responses are partly IL-12-dependent**. PBMC were cultured in the presence of an IL-12 neutralizing or isotype antibody, stimulated as indicated, and analyzed by flow cytometry. The relative percentage of IFN-γ^+^ cells among the CD4^+^ (*n* = 7), CD8^+^ (*n* = 7), γδ TCR^+^ (*n* = 7), CD161^+^Vα7.2^+^ (*n* = 4), and CD3^−^CD56^+^ (*n* = 7) lymphocyte populations is shown. The percentage of IFN-γ^+^ cells in isotype-treated PBMC stimulated with *S. aureus* or SEA is set to 1, and the percentage of IFN-γ^+^ cells in α-IL-12-treated cultures is relative to the isotype. Bars show medians with interquartile range.

We have previously shown that *S. aureus* 161:2 induce potent activation of T_H_-cells and T_regs_ (12, 29). Here, we investigated if CFS derived from *S. aureus* 161:2 could also activate innate T cells like γδ T cells and MAIT cells as well as NK cells in addition to CD4^+^ and CD8^+^ T cells (gating strategies for the identification of T and NK cell populations are displayed in Figure 1). *S. aureus*-CFS readily induced IFN-γ expression in the γδ T cell-, MAIT cell-, and NK cell populations, in addition to the CD4^+^ and CD8^+^ T cell populations (Figures 2A,B). Enterotoxins produced by *S. aureus* are classically thought to polyclonally activate a large number of adaptive T cells. However, we could show that purified SEA also induces IFN-γ expression in unconventional T cells and NK cells (Figure 2A). Considering that IL-12 is involved in the differentiation on naive T cells into T_H_1-cells and stimulates the production of IFN-γ from T cells and NK cells (30), we further investigated whether IL-12 was involved in *S. aureus*- and SEA-mediated IFN-γ induction. Indeed, there was a reduction, but not complete abrogation, in the percentage of IFN-γ^+^ cells in all investigated populations when IL-12 was neutralized (Figure 3).

### Lactobacilli-CFS Dampens Stimulated IFN-γ Responses in CD4^+^ and CD8^+^ T Cells, γδ T Cells, MAIT Cells, and NK Cells

**Figure 4:**
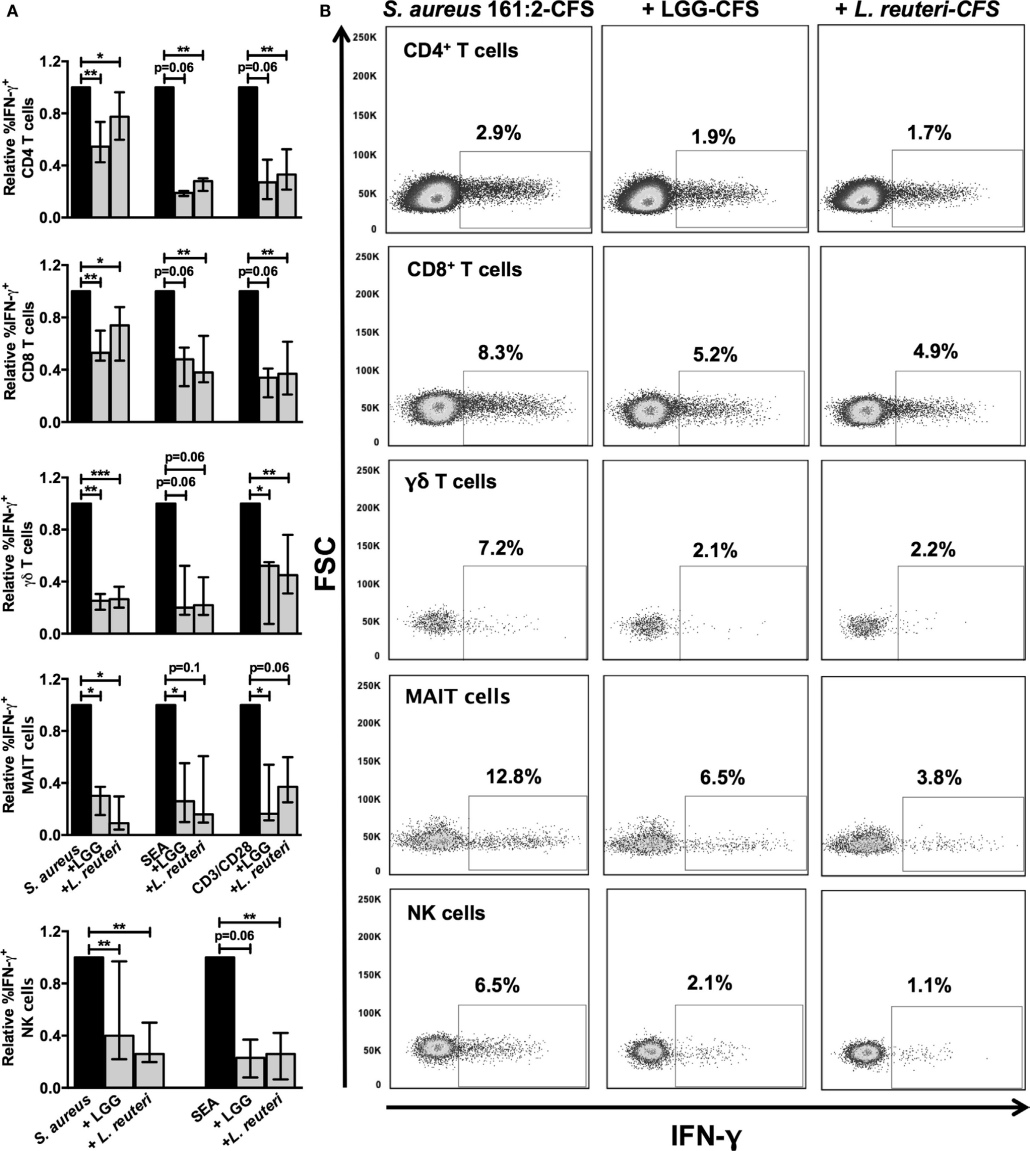
**Lactobacilli-CFS dampens *S. aureus*, SEA, and CD3/CD28-induced IFN-γ responses**. PBMC were stimulated as indicated in combination with either LGG-CFS or *L. reuteri*-CFS and analyzed by flow cytometry. **(A)** The relative percentage of IFN-γ^+^ cells among the CD4^+^ (*n* = 6–14), CD8^+^ (*n* = 6–15), γδ TCR^+^ (*n* = 5–15), CD161^+^Vα7.2^+^ (*n* = 4–7), and CD3^−^CD56^+^ (*n* = 6–15) lymphocyte populations is shown. The percentage of IFN-γ^+^ cells in the presence of lactobacilli-CFS is relative to the percentage of IFN-γ^+^ cells in the absence of lactobacilli-CFS, which is set to 1. **(B)** Examples of IFN-γ stainings of the cell populations described in **(A)**. Bars show medians with interquartile range.

We previously observed that several species of lactobacilli can dampen *S. aureus*-induced IFN-γ expression by PBMC (12), and that children who are co-colonized by lactobacilli and *S. aureus* have a dampened cytokine response *in vitro* compared to children who are colonized with *S. aureus* in the absence of lactobacilli (11). We therefore investigated whether two different lactobacilli strains, LGG and *L. reuteri* DSM 17938, both commonly used as probiotics (31–33), could modulate *S. aureus*-induced IFN-γ responses by both conventional and unconventional T cells as well as NK cells. Both LGG-CFS and *L. reuteri-*CFS dampened the *S. aureus*-induced IFN-γ response in all cell types investigated (Figures 4A,B). Interestingly, lactobacilli-CFS was also able to reduce the percentage of IFN-γ expressing cells induced by SEA and CD3/CD28-beads (Figure 4A). Lactobacilli-CFS alone did not induce expression of IFN-γ (Figure S1 in Supplementary Material).

### Lactobacilli-CFS Reduces *S. aureus*-Induced Proliferation and Cytotoxic Potential

**Figure 5:**
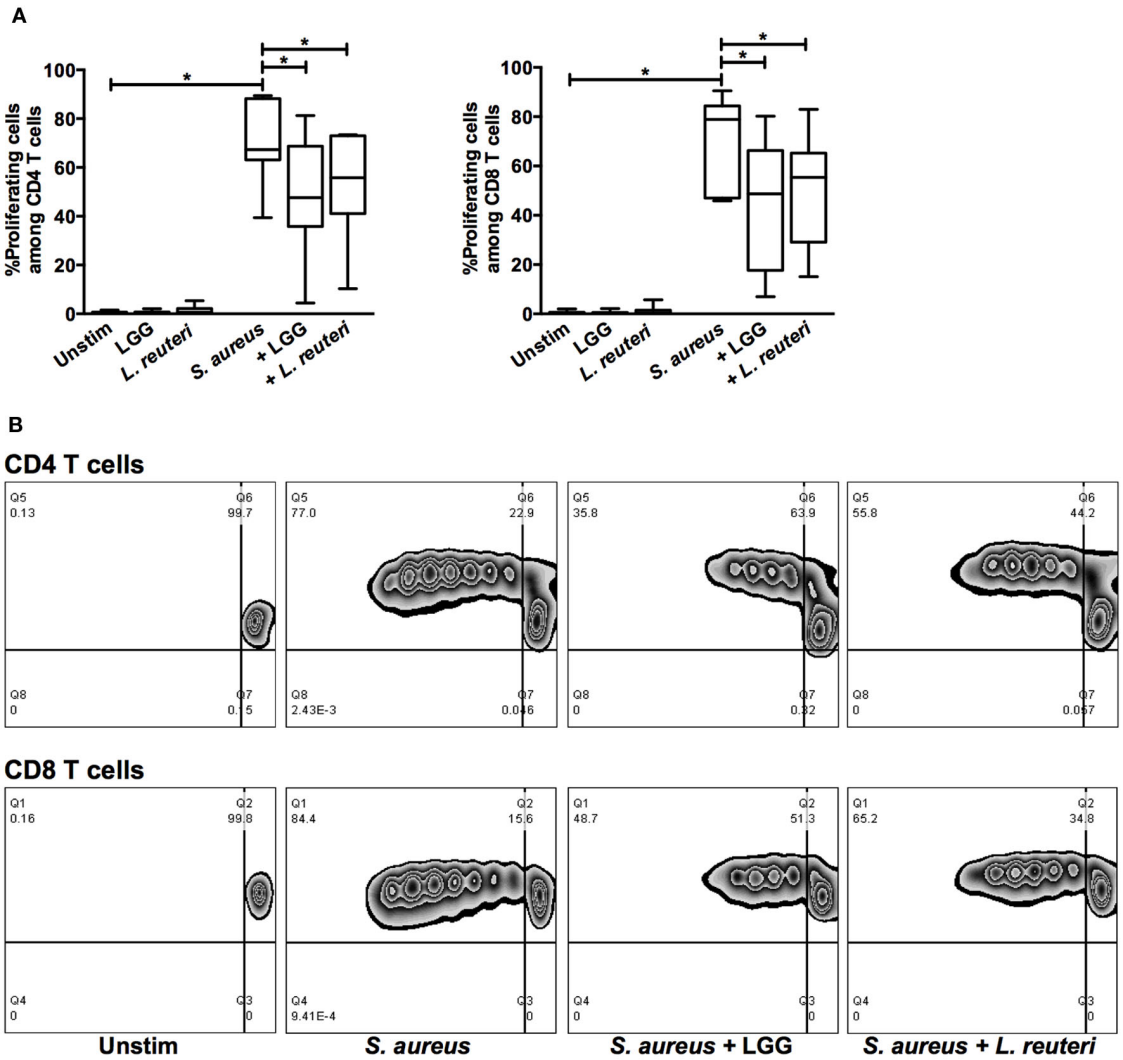
***S. aureus*-induced proliferation of CD4^+^ and CD8^+^ T cells is modulated by lactobacilli-CFS**. Proliferation was assessed with CellTrace™ Violet after 5 days of stimulation with *S. aureus* 161:2-CFS and/or lactobacilli-CFS and analyzed by flow cytometry. **(A)** The percentage of proliferating cells among the CD4^+^ (*n* = 7) and CD8^+^ (*n* = 7) T cell populations. **(B)** Representative stainings of proliferating CD4^+^ or CD8^+^ T cells are shown. Boxes cover data values between the 25th and 75th percentiles, with the central line as median and error bars showing minimal and maximal values.

**Figure 6:**
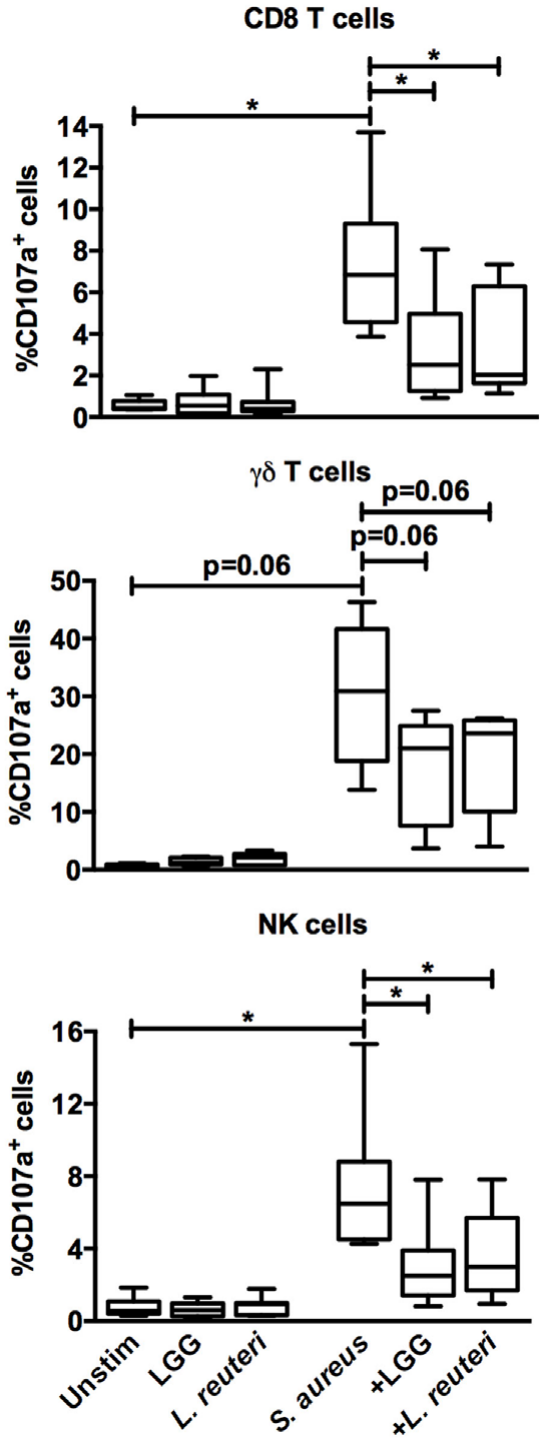
***S. aureus*-induced cytotoxic potential in CD8^+^ T cells, γδ T cells, and NK cells is reduced by lactobacilli-CFS**. PBMC were stimulated for 18 h in the presence of α-CD107a antibodies and the percentage CD107a^+^ cells among the CD8^+^ (*n* = 7), γδ TCR^+^ (*n* = 5), and CD3^−^CD56^+^ (*n* = 7) lymphocyte populations was analyzed by flow cytometry. Boxes cover data values between the 25th and 75th percentiles, with the central line as median and error bars showing minimal and maximal values.

Based on these results, we further investigated the modulatory effect of LGG-CFS and *L. reuteri*-CFS on *S. aureus*-induced lymphocyte activation. *S. aureus*-CFS induced both notable proliferation of CD4^+^ and CD8^+^ T cells (Figures 5A,B) and cytotoxic potential in CD8^+^ T cells, γδ T cells, and NK cells, measured as expression of CD107a, which is exposed at the surface of cytotoxic cells during the release of granular contents (Figure 6). In accordance with the IFN-γ expression, lactobacilli-CFS could dampen or tended to dampen both proliferation and degranulation induced by *S. aureus*-CFS (Figures 5A,B and 6).

### Lactobacilli-Mediated Dampening of IFN-γ Responses Is IL-10-Independent

**Figure 7:**
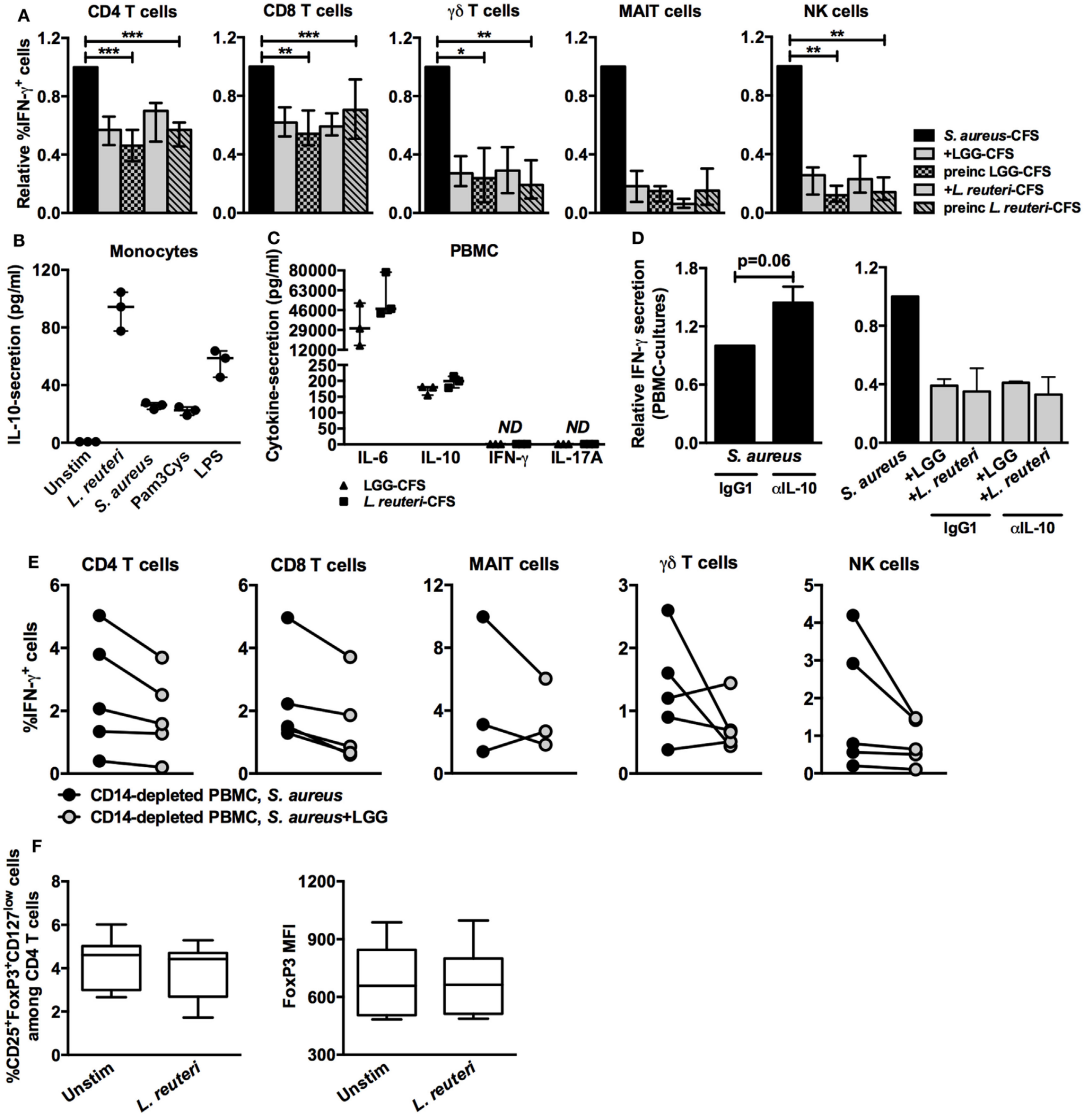
**Lactobacilli-mediated inhibition of IFN-γ responses is not dependent on monocyte-derived IL-10**. **(A)** The percentage of IFN-γ^+^ cells among the CD4^+^ (*n* = 9–14), CD8^+^ (*n* = 9–14), γδ TCR^+^ (*n* = 4–9), CD161^+^Vα7.2^+^ (*n* = 4), and CD3^−^CD56^+^ (*n* = 6–13) lymphocyte populations. Briefly, PBMC were preincubated with lactobacilli-CFS for 5 h and thereafter washed and stimulated with *S. aureus*-CFS. The percentage of IFN-γ^+^ cells in the presence of lactobacilli-CFS is relative to the percentage of IFN-γ^+^ cells in the absence of lactobacilli-CFS, which is set to 1. **(B)** Secreted levels (picogram per milliliter) of IL-10 from stimulated CD14^+^ cell cultures measured by ELISA (*n* = 3). **(C)** Secreted levels of IL-6, IL-10, IFN-γ, and IL-17A in PBMC cultures (*n* = 3) measured by ELISA. ND, not detected. **(D)** The relative IFN-γ secretion in PBMC cultures stimulated in the presence or absence of an α-IL-10 antibody. Left: levels of IFN-γ secretion in PBMC cultures stimulated with *S. aureus* is set to 1, and the levels of IFN-γ secretion in α-IL-10-treated PBMC cultures stimulated with *S. aureus*-CFS are shown as relatives to 1. Right: levels of IFN-γ secretion in PBMC cultures stimulated with *S. aureus* in the absence of lactobacilli-CFS is set to 1, and the levels of IFN-γ secretion in α-IL-10-treated PBMC cultures stimulated with *S. aureus*-CFS in the presence of lactobacilli-CFS are shown as relatives to 1. **(E)** The percentage of IFN-γ^+^ cells among the CD4^+^ (*n* = 5), CD8^+^ (*n* = 4), γδ TCR^+^ (*n* = 5), CD161^+^Vα7.2^+^ (*n* = 3), and CD3^−^CD56^+^ (*n* = 5) lymphocyte populations in monocyte-depleted PBMC cultures after stimulation with *S. aureus*-CFS in the absence or presence of LGG-CFS. **(F)** The percentage of CD25^+^FoxP3^+^CD127^low^ cells and the MFI of FoxP3 in CD4^+^ T cells after stimulation with *L. reuteri*-CFS. Bars show medians with interquartile range. Boxes cover data values between the 25th and 75th percentiles, with the central line as median and error bars showing minimal and maximal values.

To investigate whether lactobacilli-CFS was required to be present continuously in the cultures in order to dampen IFN-γ responses, we preincubated PBMC cultures with LGG-CFS or *L. reuteri*-CFS, which was followed by extensive washing prior to stimulation with *S. aureus*-CFS. Interestingly, preincubation dampened IFN-γ expression in all cell types (Figure 7A). As lactobacilli are known to induce the production of IL-10, an important immune regulatory cytokine, we speculated that lactobacilli-induced monocyte-derived IL-10 was responsible for the reduction in IFN-γ. Lactobacilli-CFS induced IL-10 secretion in cultures of purified CD14^+^ monocytes (Figure 7B) and in PBMC cultures (Figure 7C). Neutralization of IL-10 tended to result in increased IFN-γ secretion from *S. aureus*-stimulated PBMC cultures but did not influence lactobacilli-mediated dampening (Figure 7D), indicating that the effect was IL-10 independent. Further, depletion of monocytes from PBMC cultures did not seem to prevent dampening of *S. aureus*-induced intracellular IFN-γ expression (Figure 7E).

Expression of the transcription factor FoxP3 is a hallmark feature of T regulatory cells. *L. reuteri*-CFS neither altered the percentage of CD25^+^Foxp3^+^CD127^low^ cells among the CD4 T cells nor affected the magnitude of FoxP3 expression (Figure 7F), indicating that T regulatory cells were not involved in lactobacilli-mediated dampening.

### Lactate Selectively Inhibits the IFN-γ Response of Unconventional T Cells and NK Cells

**Figure 8:**
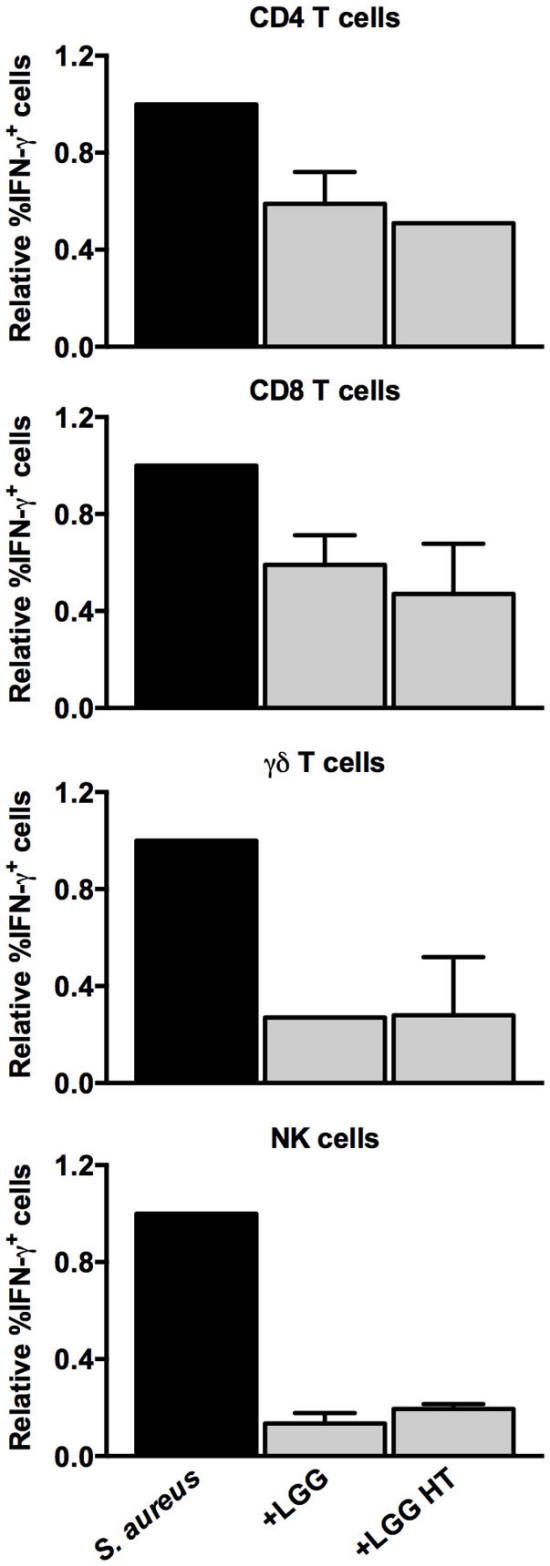
**The dampening capacity of lactobacilli-CFS is not affected by heat treatment**. The relative percentage of IFN-γ^+^ cells among the CD4^+^, CD8^+^, γδ TCR^+^, and CD3^−^CD56^+^ lymphocytes populations (*n* = 3–4) after stimulation with *S. aureus*-CFS alone or in combination with regular or heat-treated (HT) LGG-CFS. The percentage of IFN-γ^+^ cells in the presence of LGG-CFS is relative to the percentage of IFN-γ^+^ cells in the absence of lactobacilli-CFS, which is set to 1. Bars show medians with interquartile range.

**Figure 9:**
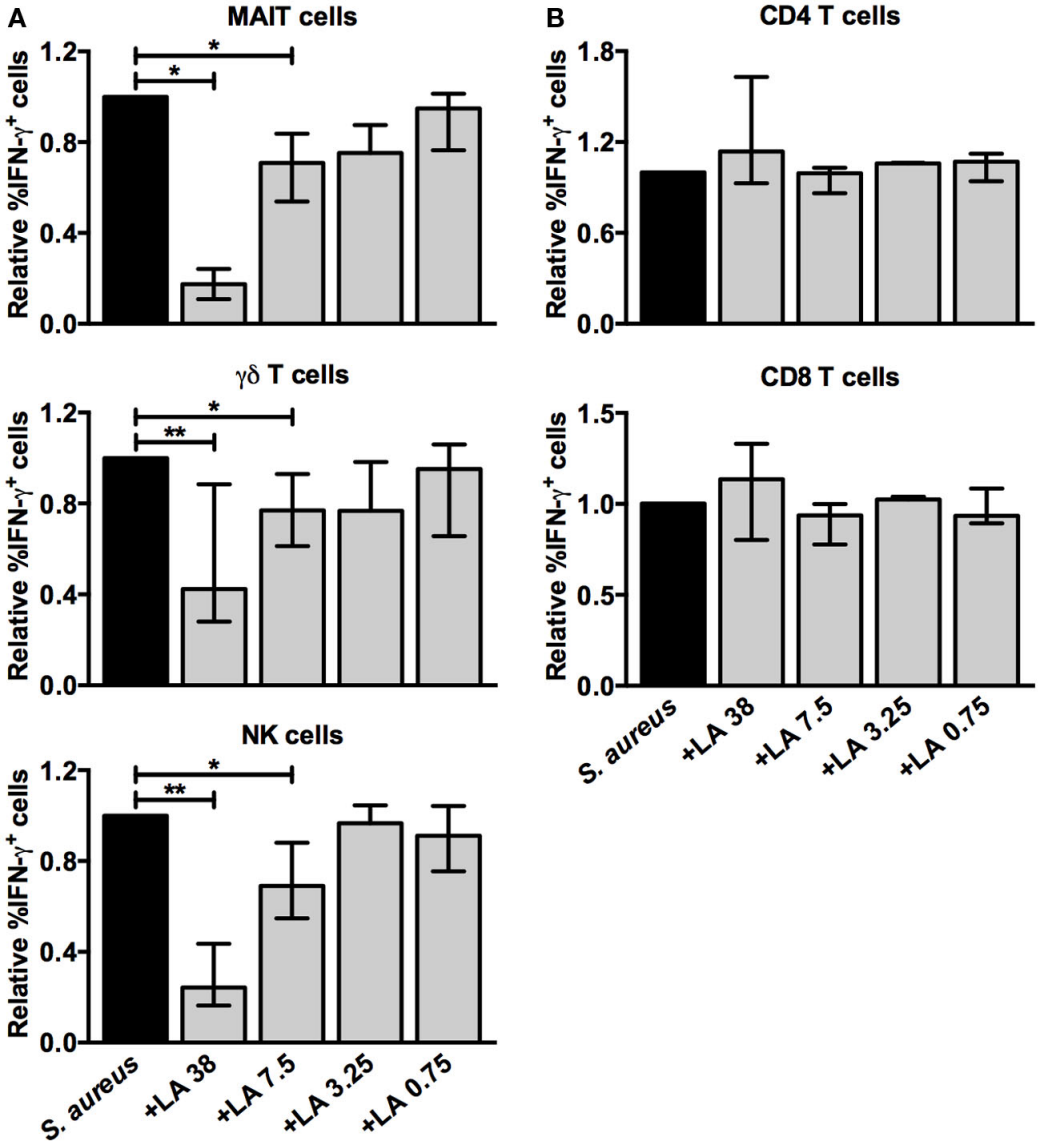
**L(+)-lactate dampens the *S. aureus*-induced IFN-γ response of γδ T cells, MAIT cells, and NK cells**. PBMC were stimulated with *S. aureus*-CFS alone or in combination with lactate (LA 38, 7.5, 3.25, or 0.75 mM). **(A)** The relative percentage of IFN-γ^+^ cells among the CD161^+^Vα7.2^+^, γδ TCR^+^, and CD3^−^CD56^+^ lymphocyte populations (*n* = 6–8). **(B)** The relative percentage of IFN-γ^+^ cells among the CD4^+^ and CD8^+^ lymphocyte populations (*n* = 6). The percentage of IFN-γ^+^ cells in the presence of lactate are relative to the percentage of IFN-γ^+^ cells in cultures stimulated with *S. aureus*-CFS alone, which is set to 1. Bars show medians with interquartile range.

To further characterize the dampening effect, we heat-treated the lactobacilli-CFS. The heat-treated LGG and regular LGG seemed to similarly reduce IFN-γ expression (Figure 8), suggesting that the effect is mediated by a heat-stable component. Lactobacilli produce heat-stable lactic acid, which is known to modulate lymphocyte-activation through acidification. Considering that the CFS used in this study were pH-neutral and that lactobacilli metabolize glucose into lactate, we hypothesized that lactate could be responsible for the observed immune modulation. First, we confirmed the presence of lactate in the CFS (Table 1). We then investigated whether commercial lactate at physiologically relevant concentrations could mimic the modulatory capacity of lactobacilli-CFS. Notably, lactate dampened the percentage of IFN-γ expressing MAIT cells, γδ T cells, and NK cells, after stimulation with *S. aureus*-CFS, in a dose-dependent manner (Figure 9A). Interestingly, CD4^+^ and CD8^+^ T cell IFN-γ expression was not affected (Figure 9B). Lactate at concentrations of 7.5 mM or below did not affect cell viability.

**Table 1:** **Concentrations of L(+)-lactate (mM)**.

	CFS^a^	Start of cell culture^b^
*L. rhamnosus* GG (LGG)	154.8 ± 7.4	3.9
*L. reuteri* DSM 17938	93 ± 2.5	2.3
*S. aureus* 161:2	ND	–

*^a^Concentrations are shown as mean ± SEM and are adjusted for the background level of the lactobacilli growth medium*.

*^b^Calculated values after dilution of CFS at the start of cell culture*.

*ND: not detected*.

## Discussion

We previously demonstrated an inverse association between early life colonization with lactobacilli and *S. aureus*, and cytokine production in infancy (11). This was further evaluated by *in vitro* studies, where lactobacilli-CFS dampened *S. aureus*-induced IFN-γ and IL-17A responses, although the responding cell types were not identified (12). In this study, we show that unconventional T cells and NK cells, in addition to conventional T cells, are strongly activated by *S. aureus*-CFS and SEA, and that CFS derived from lactobacilli is able to dampen *in vitro* activation of these cell types. Our data suggest that molecules present in the lactobacilli-CFS act directly on lymphocytes to reduce the activation since a shorter pre-stimulation of PBMC was enough to reduce subsequent activation, and because the absence of APC or APC-derived IL-10 did not prevent lactobacilli-mediated dampening. Finally, we show that lactate is a possible modulator of unconventional T cell and NK cell activation.

It is well established that SE are superantigens that activate large numbers of T cells through cross-linking of the TCR variable α or β-chains with MHC class II molecules (7–9). However, bovine CD4^+^ T cells recognize a limited number of additional *S. aureus*-antigens, implying that the mode of *S. aureus*-induced activation extends beyond enterotoxins (34). Despite the obvious impact on T cells, *S. aureus*-induced activation of T cells is understudied and mostly conducted in mice (9). Recently, we could show that *S. aureus* 161:2-CFS induced human CD4^+^FoxP3^+^ cells and promoted a diverse phenotype with the production of regulatory and pro-inflammatory cytokines (29), illustrating the complex interrelationship between *S. aureus* and the immune system. We now extend these findings and show that *S. aureus* 161:2-CFS and SEA activate and induce IFN-γ expression in human γδ T cells, MAIT cells, and NK cells, in addition to CD4^+^ and CD8^+^ T cells. Further, *S. aureus*-CFS induced both proliferation and degranulation.

To the best of our knowledge, MAIT cell responsiveness toward SE has not yet been evaluated. In normal settings, MAIT cells respond to bacteria-derived organic compounds originating from the riboflavin biosynthetic pathway in an MR1 and APC-dependent manner (35, 36). Like many bacteria, *S. aureus* uses the riboflavin pathway and promotes the activation of MAIT cells (37, 38). However, MAIT cells were not activated by CFS derived from the enterotoxin-negative *S. aureus* strain 139:3 (unpublished observation), indicating that enterotoxins are the main factors in *S. aureus* 161:2-mediated activation of the MAIT cells in our setting. Indeed, pure SEA induced activation of MAIT cells.

The activation of γδ T cells and NK cells involves antigen-presenting or accessory cells. Monocyte-derived IL-12 is a key cytokine in *S. aureus*-mediated NK cell activation (39, 40). NK cells also respond to SEA in an IL-12-dependent manner (41). Indeed, when neutralizing IL-12, the IFN-γ response toward both *S. aureus*-CFS and SEA was clearly reduced, in particular for the γδ T cells and NK cells, but also for the MAIT cells (not significant).

The main subpopulation of γδ T cells in peripheral blood primarily responds to bacterial species capable of producing the isoprenoid precursor (*E*)-4-hydroxy-3-methyl-but-2-enyl pyrophosphate (HMB-PP). This metabolite is not produced by *S. aureus* (38), and therefore it is interesting to note that γδ T cells respond to both *S. aureus*-CFS and SEA. Upon SEA inhalation, αβ T cell responses are crucial for subsequent activation of innate cells, including γδ T cells and NK cells, in mice (42). This could suggest that the activation of unconventional T cells and NK cells are secondary when stimulating PBMC with *S. aureus*-CFS or SEA. However, purified bovine γδ T cells are directly activated by SEA and SEB (43). Finally, SE have been shown to interact with additional receptors, including CD28, which confers co-stimulatory signals upon binding to CD80/CD86 on APC (44), indicating additional modes of enterotoxin-induced T cell activation.

Whole lactobacilli are described to induce both immune activation and regulation in a species-specific manner (17, 39, 45–47). Also, live and heat-treated cells of the same lactobacilli strain may differently affect immune cells (48). In addition, the capacity to activate and modulate human blood APC was similar for LGG soluble factors and whole bacteria (49). However, we have previously shown that lactobacilli-CFS from several different species all acted immune regulatory without activating the lymphocyte compartment (12). Here, we studied how CFS from two strains (LGG and *L. reuteri* DSM 17938) influenced activation of the T and NK cell compartments. Indeed, stimulation of PBMC with lactobacilli-CFS did not induce any T or NK cell activation, in terms of proliferation, degranulation, or IFN-γ production. Instead, the percentage of IFN-γ^+^ cells was dampened by lactobacilli-CFS upon activation regardless of the stimulus used. Furthermore, CD4^+^ and CD8^+^ T cell proliferation, induced by *S. aureus*, was dampened by both LGG and *L. reuteri*. Whole lactobacilli and lactobacilli-CFS have been shown to suppress proliferation of human CD4^+^ and CD8^+^ T cells without altering T cell cytokine production. This was mediated by lactobacilli-induced inhibition of the monocarboxylate transporter MCT-1 (50), through which compounds can mediate changes in T cell growth without affecting cytokine expression (51). However, the prominent dampening of IFN-γ observed in the present study suggests other modes of regulation. Mouse CD4^+^ T cell proliferation was shown to be reduced by *L. gasseri*-RNA in a MyD88-dependent manner (52), and *L. helveticus* inhibits the proliferation of LPS-stimulated murine T cells through the reduction of phosphorylation of c-Jun N-terminal kinase (JNK), which halters the cell cycle at the G2/M phase and thereby prevents cell cycle progression (53).

The lactobacilli-CFS also hampered cytotoxic potential in CD8^+^ T cells, γδ T cells, and NK cells. To our knowledge, this is the first time lactobacilli have been shown to influence CD107a expression in cytotoxic lymphocytes.

To investigate whether dampening of lymphocyte activation required a continuous presence of lactobacilli-derived factors, we preincubated PBMC with the lactobacilli-CFS, followed by extensive washing before stimulation with *S. aureus*-CFS or SEA. Also, in this setup, both LGG-CFS and *L. reuteri*-CFS were able to suppress the IFN-γ response to the same extent as if continuously present, showing that factors present in the lactobacilli-CFS alter the capacity of the cells to respond toward later microbial stimulation. Lactobacilli have been shown to induce suppressor of cytokine signaling (SOCS), a negative regulator of pro-inflammatory cytokines, in human primary macrophages (54) and in hepatocellular carcinoma cells, which were then desensitized to subsequent LPS-stimulation (55). If lactobacilli are able to induce SOCS-expression also in human T cells remains to be investigated.

Several studies have attributed the immune-modulatory potential of lactobacilli to their induction of IL-10 production by immune cells (13, 14, 24). Niers et al. showed that lactobacilli inhibit PHA-induced type 2 cytokines, partially *via* IL-10 induction (56). However, the lactobacilli-mediated dampening of IFN-γ responses observed in this study was IL-10-independent. In fact, the capacity of several lactobacilli strains to induce IL-10 in PBMC cultures varies to a high degree, yet all seem to have the ability to similarly dampen IFN-γ (our unpublished data). Further, strains of *L. plantarum* have been shown to ameliorate colitis also in IL-10-deficient mice (57, 58).

The sensing of, and signaling induced by, lactobacilli require further investigation. As reviewed by Yan and Polk, a LGG-derived protein, p40, has been attributed immune-modulatory functions, through the inhibition of epithelial cell apoptosis and inhibition of innate cytokine production (27). Heat treatment of LGG-CFS did not alter the capacity to dampen the IFN-γ response, implying that a heat-sensitive peptide/protein did not mediate dampening in our setting.

Lactobacilli species are known lactic acid-producing bacteria. Fisher et al. reported that lactic acid inhibits antigen-specific CD8 T cell cytotoxicity and that this effect was connected to acidification (59), while others reported increased CTL activation *in vitro*, but the opposite pattern *in vivo* (60). Still, the dampening nature of the lactobacilli-CFS used in this study was not due to the acidification of the cell medium, as the CFS were pH-neutralized before added to cell cultures. We therefore investigated whether lactate, which does not acidify the cell medium, could be a possible factor contributing to the suppression of IFN-γ responses. Indeed, lactate was present in the lactobacilli-CFS at physiological concentrations in the cell cultures, and commercial lactate suppressed the IFN-γ response of MAIT cells, γδ T cells, and NK cells in a dose-dependent manner. Intriguingly, tumor-derived lactate has been shown to inhibit NK cell activation and cytotoxicity (61). The responses exerted by activated T cells depend on alterations in the metabolic status, for example, in the glycolysis with lactic acid as an end product (62). This could, in part, explain why lactate acts differently on distinct types of T cells. Also, the metabolic activity of naive and memory T cells differs, indicating that the composition, for example, memory:naive ratio of the individual lymphocyte populations examined, could influence kinetics and subsequent activation (62, 63). Even though our data are limited to IFN-γ expression, they suggest that lactate could contribute to lactobacilli-mediated modulation of immune activation. However, as lactate did not affect conventional T cell activation in our setting, other factors must be responsible for the lactobacilli-mediated dampening in these cell types. We conclude that lactobacilli-CFS contain unidentified factors capable of dampening T cell and NK cell activation, and that lactate additionally dampens the activation of unconventional T cells and NK cells, which are supported by the more pronounced dampening of IFN-γ in these cell populations compared to conventional T cells.

Even though whole lactobacilli induce pro-inflammatory responses in certain settings, *in vivo* administration of lactobacilli reduces inflammatory phenotypes. Pre- and postnatal lactobacilli supplementation is associated with reduced TLR2 responsiveness during childhood (64) and protection against allergic inflammatory responses (2), indicating that lactobacilli can modulate peripheral immunity. Further, lactobacilli administration reduces viral infections in preterm infants (65). Live lactic acid-producing bacteria and their bacterial DNA reduce SEA- and allergen-induced IL-4 and IL-5 PBMC responses from both healthy and allergic individuals (3, 4). An aberrant microbiota has been associated with immune-mediated diseases, such as inflammatory bowel disease and allergy (66–68). An inverse association between lactobacilli-colonization and development of allergic disease has been reported (1, 69, 70), suggesting that lactobacilli species are important modulators of immunity. The observed dampening of pro-inflammatory responses by lactobacilli-derived factors might contribute to the protective effect of lactobacilli when present as a gut commensal or used as supplement.

In this study we extend the *in vitro* characterization of PBMC responses to *S. aureus* 161:2 and SEA, showing that in addition to potent activation of CD4^+^ and CD8^+^ T cells, also unconventional T cells and NK cells are activated. Further, we show that all responses induced by *S. aureus*-CFS were dampened by soluble factors derived from probiotic LGG and *L. reuteri* DSM 17938. The immune-modulatory effect of lactobacilli-derived factors *in vitro* provides not only a link to the beneficial effects of lactobacilli supplementation and early life colonization on immune dysregulation and immune-mediated diseases but also suggests that lactobacilli could modulate microbe-induced immune responsiveness *in vivo*.

## Author Contributions

MJ, SB, MMF, and ES-E conceived and designed the study and also wrote the paper. MJ, SB, MMF, KQ, MSC, JB, ME, and ES-E designed or/and performed laboratory experiments. MJ, SB, MMF, and KQ analyzed and/or finalized the data. MJ and SB performed statistical analysis. MJ and SB contributed equally to this work.

## Conflict of Interest Statement

The authors declare that the research was conducted in the absence of any commercial or financial relationships that could be construed as a potential conflict of interest.
