# Supplementary material for: Probiotic Lactobacilli Modulate Staphylococcus aureus-Induced Activation of Conventional and Unconventional T cells and NK Cells
Source: Front Immunol. 2016 Jul 11;7:273. doi: 10.3389/fimmu.2016.00273 (PMC4939411; doi:10.3389/fimmu.2016.00273)
Supplement: Supplementary file 1 [file image_1.pdf]

**SUPPLEMENTARY FIGURES**

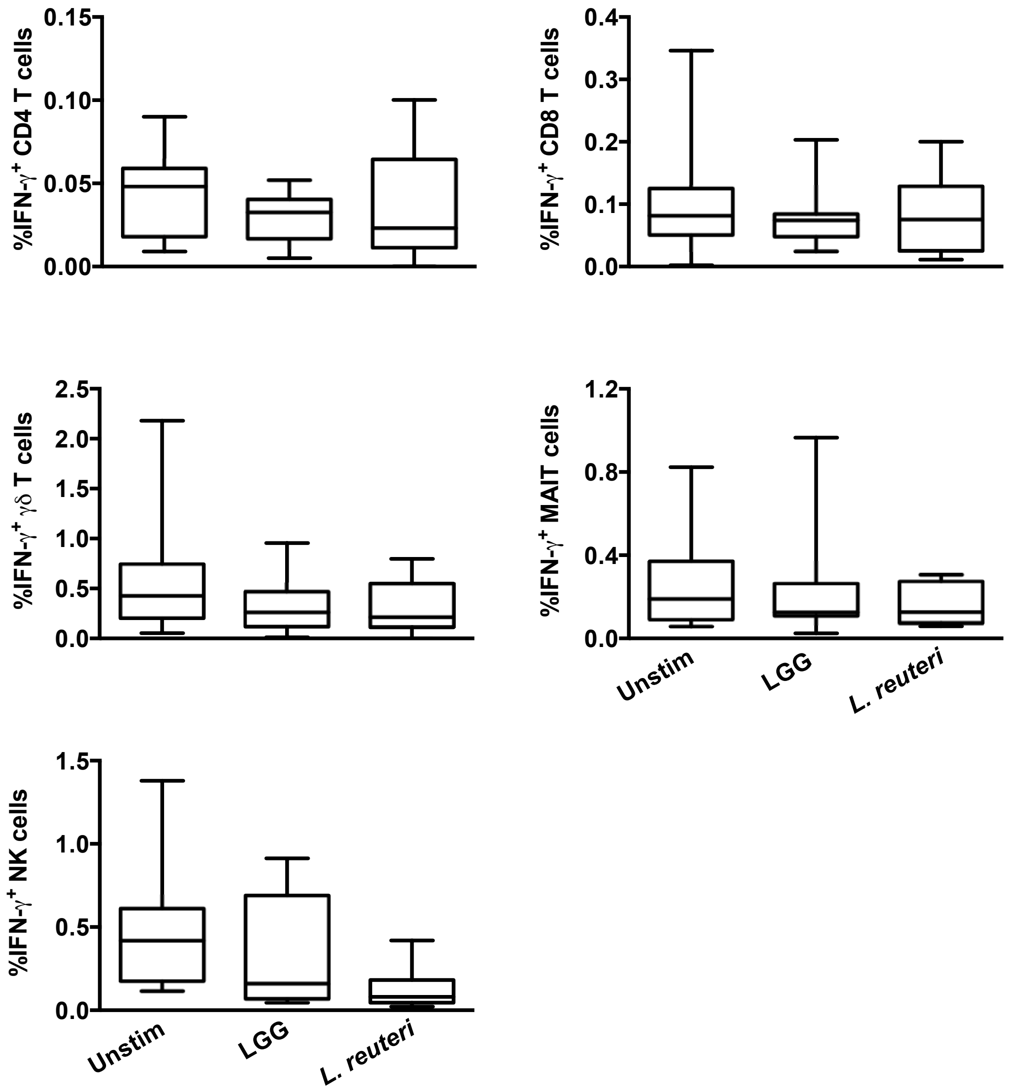


**SUPPLEMENTARY FIGURE 1. Lactobacilli-CFS does not induce intracellular IFN-γ expression.**

The percentage of IFN-$\gamma^+$ cells among the $CD4^+$ (n=10-17), $CD8^+$ (n=10-18), γδ $TCR^+$ (n=8-15), $CD161^+V\alpha7.2^+$ (n=6-8) and $CD3^-CD56^+$ (n=10-17) lymphocyte populations after 24 hour stimulation with LGG-CFS or *L. reuteri*-CFS. Boxes cover data values between the $25^{th}$ and $75^{th}$ percentiles, with the central line as median.
